# Supplementary material for: Identification of the trail-following pheromone receptor in termites
Source: eLife. 2025 Aug 19;13:RP101814. doi: 10.7554/eLife.101814 (PMC12364489; doi:10.7554/eLife.101814)
Supplement: Figure 6—source data 1. [file elife-101814-fig6-data1.docx]

Nucleotide and protein sequences of PsimOR14

***PsimOR14***

ATGATTCGATCAAAGAGAAAGGAGAGCCAAGCAAACGAAACAAAACACACATTAACAAGTGAAGAGCAACCTTCTGATTGTGACGTGAAGATCATGACACTCAGTATTATGCTGAATGCGGCTGGCCTCCTACCTCCAGCCAAATCGTCATCATTGATCAGACTGGCCTACAAAGTATTTGTAGTATTTATTCACATACTTTTCGTCTTAACGCTGATAGGACAGATAATGGCAGTAGTGGTTTACTGGGGAGACATTCCTCTAATTGCAACCACAATAAGCTTGATGACTAGTCTGATTGGATCGATGAGTTCATCCATAAATTTTCTTCTAAACAGAAAGAAGTACATGCGTCTTGCGGACACGTTGAAAACAGAATTTGTTGCCAAATTGAAATCAAAATATATCAAAATTATTTTAAATGCTGAACGTCAGGTTGTATTCTGTGGGATACTCGTATGTATTGTAGCTGTATGTATTGGATTTATTTGGATAGTCGTGCCATTTTTAAGTACCAACACCCCATTTGACTTTGCAAATGAAAAAAGTGTCAAAGAAGGAAGCCGCATGGAGGAATTAATTCTTGTGATGTGGCTCCCTTCTAAATTTGAACAGTCCCCTCAATTTGAAATAATAGTTTTTTTACAAATCTTTGTCGTAACGTTTGCATTAGCAATGATCTATTCAGTTGATATGATGTTACTATCTCTGATGAGCCACGCTGCTGCACAGTTCAGGGTTTTGAATGCCATGCTGAATGACATGCACGAAAATGTTCGTGAAGACGAGATTCACAGAACAAGAAACATGGCTTCATTGGTCACTGGCACTGACATCTCGTACATGGAGTTCTCTTCTACCAATTCTTGGAATGGAAACACAGAGCATTCTGGAAGCGCTGGTGTTGAGTTGGACAGCCTAAAAAATGAAGACTGTGAAGAAGATCCTGTCCGACAGTACCTCGTTGAGTGCATTAGATATCACCAGGCTGTAATTGAGTTTGTTGACCAACTGAACGAGGTGTTCGGCGCAGTGAGCTTCGTGAAGATGCTTGACTGGCCTTTTGCGATTTGTATGACAGGATTTCAGTTGACACAGACTGTAGAGAGCCAGGAGGATTTACTTAAATTCATCTCCCTGTTTGCTGGGGTTGTATATCTAATAATCTCTTACATTTGGTTCGGACAGCAAGTAATTGACGAGAGCGAGGAAGTAGCAACAGCGTTGTACAGCACTGACTGGTACAACCAGGCACCAGGGTTCAAACGTCTGCTGCCTGTAGCCATCATGCGGGCTTCGAATTCTGTCAAAGTTAAAGCTGGGGTGTTCTTTGACATGTCTTGCGTCACGTTAGCTTCGATTATGAATGCATCTTACACGTATTTTATGATGCTAATTCATCTACACGACTCCTAA

**PsimOR14**

MIRSKRKESQANETKHTLTSEEQPSDCDVKIMTLSIMLNAAGLLPPAKSSSLIRLAYKVFVVFIHILFVLTLIGQIMAVVVYWGDIPLIATTISLMTSLIGSMSSSINFLLNRKKYMRLADTLKTEFVAKLKSKYIKIILNAERQVVFCGILVCIVAVCIGFIWIVVPFLSTNTPFDFANEKSVKEGSRMEELILVMWLPSKFEQSPQFEIIVFLQIFVVTFALAMIYSVDMMLLSLMSHAAAQFRVLNAMLNDMHENVREDEIHRTRNMASLVTGTDISYMEFSSTNSWNGNTEHSGSAGVELDSLKNEDCEEDPVRQYLVECIRYHQAVIEFVDQLNEVFGAVSFVKMLDWPFAICMTGFQLTQTVESQEDLLKFISLFAGVVYLIISYIWFGQQVIDESEEVATALYSTDWYNQAPGFKRLLPVAIMRASNSVKVKAGVFFDMSCVTLASIMNASYTYFMMLIHLHDSX
